# *Leonurus japonicus* Houtt Attenuates Nonalcoholic Fatty Liver Disease in Free Fatty Acid-Induced HepG2 Cells and Mice Fed a High-Fat Diet

**DOI:** 10.3390/nu10010020

**Published:** 2017-12-25

**Authors:** Mi-Ra Lee, Kwang Il Park, Jin Yeul Ma

**Affiliations:** Korea Institute of Oriental Medicine, 70 Cheomdan-Ro, Dong-Gu, Daegu 41062, Korea; withme@kiom.re.kr (M.R.L.); kipark@kiom.re.kr (K.I.P.)

**Keywords:** free fatty acids, HepG2 cells, high-fat diet, obesity, *Leonurus japonicus*, nonalcoholic fatty liver disease

## Abstract

We investigated the effects of a *Leonurus japonicus* ethanol extract (LJE) on nonalcoholic fatty liver disease (NAFLD). An in vitro model of hepatic steatosis was treated with 1 mM free fatty acid (FFA) in HepG2 cells. An in vivo NAFLD model was established using C57BL/6 mice fed a high-fat diet (HFD) and administered LJE (100 or 200 mg/kg) orally for 14 weeks. LJE treatment suppressed lipid accumulation and intracellular triglyceride levels significantly in a concentration-dependent manner in HepG2 cells. Moreover, LJE significantly reduced the expression of sterol regulatory element binding protein 1-c, and its downstream genes, which are associated with lipogenesis, in HepG2 cells. In HFD-fed mice, LJE treatment decreased body weight significantly and decreased serum alanine transaminase levels to normal values, concurrent with a decrease in hepatic lipid accumulation. Furthermore, LJE supplementation ameliorated insulin sensitivity by decreasing serum glucose and insulin levels. LJE improved hepatic steatosis by increasing the expression of phosphorylated AMP-activated protein kinase and peroxisome proliferator-activated receptor-α in HFD-fed mice and FFA-treated HepG2 cells. The results suggested that LJE might be a potential therapeutic agent to treat NAFLD.

## 1. Introduction

Nonalcoholic fatty liver disease (NAFLD) is a chronic liver disease closely associated with metabolic diseases [1]. NAFLD represents a wide range of pathological liver damage, from simple steatosis to steatohepatitis associated with inflammation, fibrosis, and cirrhosis [2,3]. The high-fat diet (HFD) has been used extensively to induce obesity and obesity-related metabolic disorders in experimental animals [4]. Long-term HFD-induced NAFLD animal models provide useful information to test the therapeutic effects of various agents, as well as to elucidate the pathogenesis of NAFLD [5,6]. Alternatively, HepG2 cells treated with free fatty acid (FFA) have been used commonly to induce the hepatic steatosis of NAFLD [7,8]. 

Pharmacological treatments for NAFLD include lipid-lowering agents (statins, fibrates), insulin sensitizers (metformin, thiazolidinediones), cytoprotective and antioxidant agents (bile acids, vitamin E), and anti-obesity drugs (orlistat) [9]. However, these drugs are not liver-specific agents and increase the risk of side effects caused by long-term ingestion. Therefore, safe and effective therapeutic agents such as natural herbal medicines are required to treat hyperlipidemia and obesity associated with NAFLD [10,11]. 

*Leonurus japonicus* Houtt. (Lamiaceae), known as motherwort, is distributed throughout Asia [12]. The aerial part of *L. japonicus* has been used as a medicinal herb for gynecological diseases, including menoxenia, dysmenorrhea, and amenorrhea. Pharmacological studies of *L. japonicus* have demonstrated various beneficial effects, including anticancer, neuroprotection, and antibiosis activities [13,14,15]. Moreover, a previous report described the effects of an *L. japonicus* aqueous extract on HFD-fed post-menopausal obese mice [16]. However, there has been no investigation of the effects of *L. japonicus* on NAFLD caused by HFD-induced obesity. 

In the present study, we investigated the pharmacological effects of an *L. japonicus* ethanol extract (LJE) on the improvement of NAFLD via in vitro and in vivo models. 

## 2. Materials and Methods

### 2.1. Preparation of L. japonicus Ethanol Extract (LJE)

*L. japonicus* (dried aerial parts) was obtained from an herbal market (Yeongcheon, Korea). Prof. Ki Hwan Bae (Chungnam National University, Daejeon, Korea) confirmed the identification of the plant. *L. japonicus* was extracted with 70% ethanol for 24 h, and concentrated and lyophilized to a powder. The LJE yield was 7.37%.

### 2.2. Cell Culture

HepG2 cells were obtained from the American Type Culture Collection (Manassas, VA, USA) and maintained in Dulbecco’s modified eagle medium containing 1% penicillin/streptomycin and 10% fetal bovine serum in an atmosphere of 5% CO_2_ at 37 °C. The FFA mixture (oleic acid/palmitic acid, 2:1) was prepared with fat-free bovine serum albumin (BSA). Untreated control cells were treated with 1% BSA. After attaining 70% confluence, HepG2 cells were cultured with FBS-free medium overnight and then exposed to 1 mM FFA containing 1% BSA, with or without LJE, for 24 h. 

### 2.3. Cell Viability

HepG2 cells were plated at 4 × 10^4^ cells/well in 96-well plates. The cells were starved and then exposed to 1 mM FFA and LJE at different concentrations (0, 250, 500, 750, or 1000 μg/mL) for 24 h. The cells were then incubated with 10 μL of MTT solution (5 mg/mL) for 4 h. The formazan crystals were dissolved in 200 μL of DMSO and the absorbance was measured at 570 nm. Each treatment was performed in triplicate. 

### 2.4. Oil Red O Staining

HepG2 cells were fixed with 10% formalin for 30 min and then stained with Oil Red O solution for 20 min. The plates were washed and the stained cells were observed under an Olympus CKX 41 microscope (Olympus, Tokyo, Japan). After extraction with 100% isopropanol, the Oil Red O content was quantified at 500 nm.

### 2.5. Fluorometric Determination of TG Content by Nile Red 

Intracellular TG contents were determined fluorometrically using the commercial reagent AdipoRed, comprising a solution of the hydrophilic stain Nile Red (Lonza, Walkersville, MD, USA). HepG2 cells were plated at 4 × 10^4^ cells/well in 96-well plates. The plates were washed and the cells were incubated with AdipoRed solution for 10 min before the fluorescence was measured at an excitation wavelength of 485 nm and an emission wavelength of 572 nm. 

### 2.6. Immunoblot Analysis

Sample proteins were extracted using RIPA lysis buffer with phosphatase and protease inhibitor cocktails. Equal amounts of protein (20 μg) were separated on 8–10% SDS-PAGE gels, and then transferred to polyvinylidene fluoride membranes. The membranes were blocked for 1 h and then probed with specific antibodies. The immunoreactive protein bands were detected using an enhanced chemiluminescence western blot detection kit and visualized using the Chemidoc™ Touch image system (Bio-Rad laboratories, Hercules, CA, USA). 

### 2.7. Analysis of mRNA Expression

Total RNA was isolated using the TRIzol reagent according to the manufacturer’s instructions. One microgram of RNA was used to synthesize cDNA. Real-time PCR was performed using an AccuPower^®^ GreenStar qPCR Master Mix (Bioneer, Daejeon, Korea) on a C1000 Touch Thermal cycler with the CFX manager software (Bio-Rad laboratories, Hercules, CA, USA). Primer sequences are shown in Table 1.

### 2.8. Animal Experiment

Five-week-old male C57BL/6 mice were obtained from Samtako BioKorea Inc. (Osan, Korea). Diet-induced obese mice were divided into four groups (*n* = 8 each) fed a normal diet (Con, 10 kcal% fat, D12450B, Research Diet, Inc., New Brunswick, NJ, USA) or an HFD (60 kcal% fat, D12492), with or without LJE (100 or 200 mg/kg, daily oral administration), for 14 weeks. Blood was collected from the portal vein, and organs were removed. Tissue samples were snap-frozen in liquid nitrogen and stored at −80 °C. The Institutional Animal Care and Use Committee of the Korea Institute of Oriental Medicine approved the protocols for the animal experiment (IACUC, KIOM-D-16-022).

### 2.9. Biochemical Assays

Serum triglycerides (TG), total cholesterol (TC), low-density lipoprotein cholesterol (LDL-C), aspartate aminotransferase (AST), alanine aminotransferase (ALT), lactate dehydrogenase (LDH), and glucose were measured by enzymatic colorimetric methods using an Erba XL-200 analyzer (ERBA diagnostics Mannheim GmbH, Mannheim, Germany). Serum insulin (ALPCO, Salem, NH, USA) and malondialdehyde (MDA, Cell Biolabs, San Diego, CA, USA) levels were determined using commercial kits. Homeostasis model assessment-insulin resistance (HOMA-IR) was calculated using the following formula: HOMA-IR = (insulin × glucose)/405. 

### 2.10. Liver Lipids, TG, and TC 

Hepatic lipids were extracted from liver homogenates using a chloroform/methanol solution (2:1), as previously described [17]. Briefly, 100 mg of liver was homogenized in 0.5 mL phosphate-buffered saline, added to 3 mL of chloroform/methanol (2:1), and vortexed vigorously for 2 h. After centrifugation, the lipid-containing phase was dried under nitrogen gas and resuspended in 100 μL Triton X-100/methanol (2:1). Hepatic TG and TC levels were analyzed with commercial kits according to the manufacturer’s protocol. 

### 2.11. Hematoxylin and Eosin Staining (HE)

Liver tissues were fixed in 10% formalin and embedded in paraffin. The tissue slices (4 μm) were then stained with hematoxylin and eosin. 

### 2.12. Statistical Analysis

All data are expressed as means ± SEM and were analyzed using GraphPad Prism 5.03 (Graph pad software, La Jolla, CA, USA). Statistical analysis was performed using analysis of variance, followed by Tukey’s post-hoc tests. Differences with *p* values less than 0.05 were considered significant.

## 3. Results

### 3.1. Cell Viability of LJE

**Figure 1:**
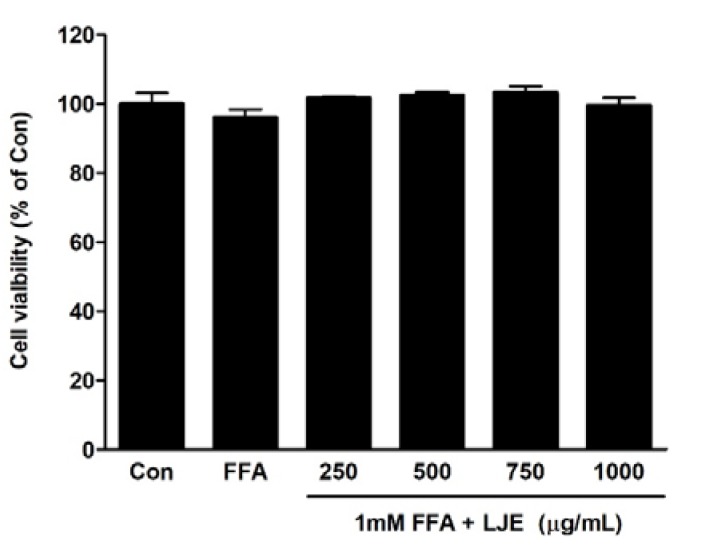
Effects of *L. japonicus* ethanol extract (LJE) on the viability of HepG2 cells. HepG2 cells were exposed to various concentrations of LJE and 1 mM free fatty acid (FFA). Data represent means ± SEM of three independent experiments.

The effects of different concentrations of LJE plus 1 mM FFA on cell viability were determined using the MTT assay. LJE treatment at a concentration of up to 1000 μg/mL and FFA for 24 h had no cytotoxic effect (Figure 1).

### 3.2. Effects of LJE on Lipid Accumulation in HepG2 Cells

**Figure 2:**
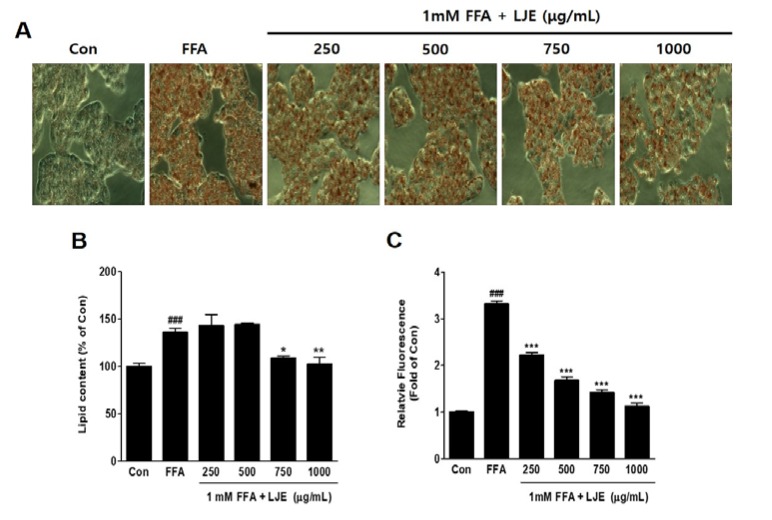
Effect of LJE on intracellular lipid accumulation in HepG2 cells. Control (Con) cells were treated with 1% fat-free bovine serum albumin. (**A**) Oil Red O staining images of HepG2 cells (magnification 200×); (**B**) Quantitative Oil Red O contents at 500 nm; (**C**) Intracellular triglyceride contents were measured by fluorescence intensity. Data represent means ± SEM. ^###^ *p* < 0.001 vs. Con; * *p* < 0.05, ** *p* < 0.01, *** *p* < 0.001 vs. FFA.

HepG2 cells were exposed to 1 mM FFA to induce hepatic steatosis, and intracellular lipid accumulation was visualized by Oil Red O staining. FFA-induced HepG2 cells showed significant increases in lipid droplets compared with the untreated BSA-control (Figure 2A,B), and hepatic steatosis was significantly inhibited by LJE at high concentrations of 750 and 1000 μg/mL (Figure 2A,B). The quantitative determination of intracellular TG content by Nile Red analysis showed a concentration-dependent decrease in the LJE-treated HepG2 cells (Figure 2C).

### 3.3. Effects of LJE on Lipogenesis in HepG2 Cells

**Figure 3:**
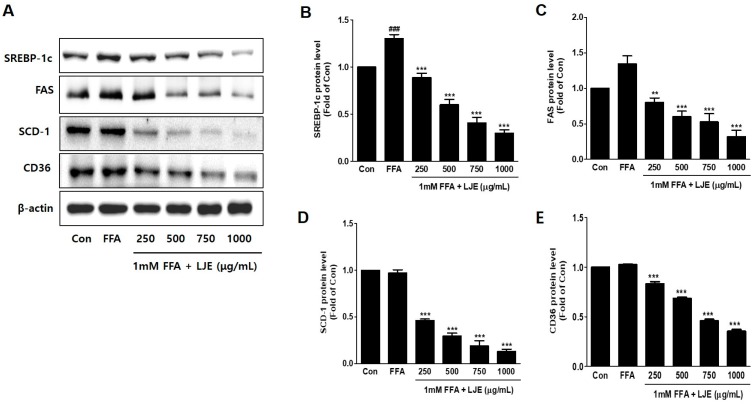

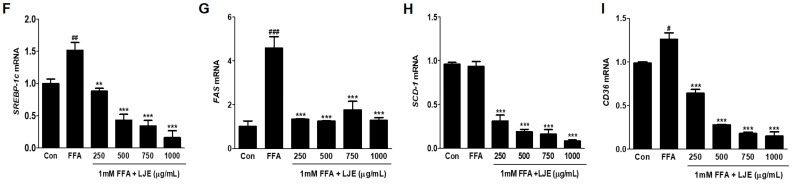
Effects of LJE on lipogenesis markers in HepG2 cells. (**A**–**E**) Immunoblot analysis; (**F**–**I**) Quantitative real-time PCR analysis. Data represent means ± SEM. ^#^ *p* < 0.05, ^##^ *p* < 0.01, ^###^ *p* < 0.001 vs. Con; ** *p* < 0.01, *** *p* < 0.001 vs. FFA.

To investigate the mechanisms underlying LJE-regulated anti-hepatic steatosis, lipogenic markers (SREBP-1c, FAS, SCD-1, and CD36) were detected by immunoblotting and real-time PCR. Immunoblotting analysis showed that SREBP-1c, FAS, SCD-1, and CD36 protein levels were significantly decreased in a concentration-dependent manner by LJE treatment compared with FFA-treated HepG2 cells (Figure 3A–E). In addition, real-time PCR analysis revealed that *SREBP-1c*, *FAS*, *SCD-1*, and *CD36* mRNA expression were also reduced in LJE-treated HepG2 cells (Figure 3F–I). These results indicated that LJE treatment downregulated lipogenesis markers in FFA-induced HepG2 cells, further suggesting that the suppression of TG accumulation is involved in the LJE-induced reduction of lipid accumulation.

### 3.4. Effects of LJE on Phosphorylation of AMP-activated Protein Kinase (AMPK) and Acetyl-CoA Carboxylase (ACC) and mRNA Expression of PPARα and CPT-1 in HepG2 Cells

**Figure 4:**
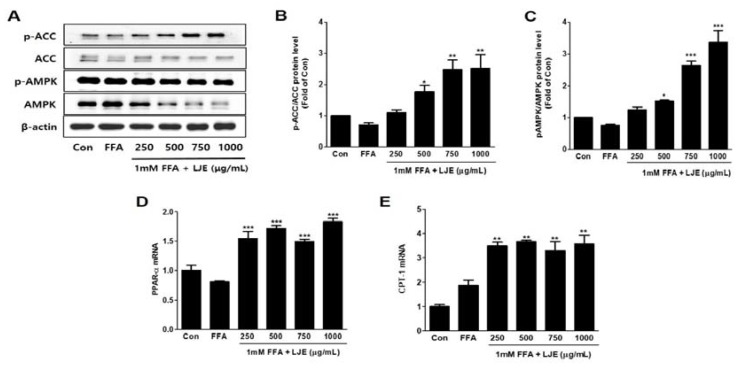
Effects of LJE on the phosphorylation of AMP-activated protein kinase (AMPK) and acetyl-CoA carboxylase (ACC) and the mRNA expression of fatty acid oxidation factors in HepG2 cells. (**A**–**C**) AMPK and ACC phosphorylation levels; (**D**,**E**) PPAR-α and CPT-1 mRNA levels. Data represent means ± SEM. * *p* < 0.05, ** *p* < 0.01, *** *p* < 0.001 vs. FFA.

LJE significantly increased the phosphorylation of AMPK and ACC, which is the direct substrate of AMPK (Figure 4A–C). *PPAR-α* and *CPT1* mRNA levels (related to β-oxidation) were also significantly increased by LJE treatment in FFA-induced HepG2 cells (Figure 4D,E).

### 3.5. Effects of LJE on Body Weight and Visceral White Adipose Tissues in HFD-Fed Mice

**Figure 5:**
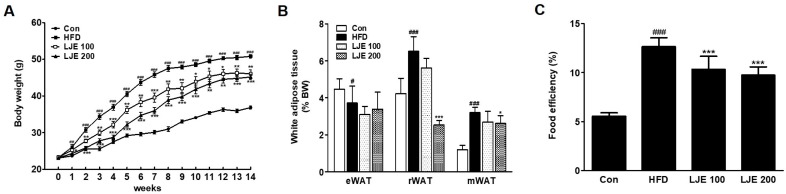
Effects of LJE on the body weight and visceral white adipose tissues in high-fat diet (HFD)-fed mice. (**A**) Body weight changes; (**B**) Visceral white adipose tissue; (**C**) Food efficiency. Con, normal control diet; HFD, high-fat diet; LJE 100, HFD plus LJE (100 mg/kg, peroral); LJE 200, HFD plus LJE (200 mg/kg, peroral). Data represent means ± SEM (*n* = 8). ^#^ *p* < 0.05, ^##^ *p* < 0.01, ^###^ *p* < 0.001 vs. Con; * *p* < 0.05, ** *p* < 0.01, *** *p* < 0.001 vs. HFD.

Figure 5 shows the changes in body weight, white adipose tissue, and food efficiency in the experimental animals. The effects of HFD on body weight gain were significantly different from week 1 of the experiment (Figure 5A). However, LJE treatment (100 or 200 mg/kg) reduced body weight gain significantly from weeks 1 and 2 compared with the HFD group, respectively (Figure 5A). The weights of visceral white adipose tissues (retroperitoneal white adipose tissue (rWAT) and mesenteric white adipose tissue (mWAT)) increased significantly in the HFD group compared with those in the control group (by 154% and 261%, respectively, Figure 5B). LJE treatment (LJE 200; 200 mg/kg) decreased rWAT (61%) and mWAT weights (8.8%) significantly compared with those in the HFD group. Food efficiency estimates the utilization of food consumed with respect to weight gain. Figure 5C shows that LJE reduced the increased food efficiency in HFD-fed mice significantly. These results showed that LJE supplementation improved the obesity-related phenotype of fat deposition in HFD-fed mice.

### 3.6. Effects of LJE on Serum Lipids, Liver Enzymes, and MDA Levels in Serum

To further investigate the preventive effects of LJE on HFD-induced liver steatosis, we measured serum biochemical indices. As shown in Table 2, mice fed an HFD exhibited significantly increased serum TC and LDL-C levels compared with mice fed a normal diet. LJE administration (100 or 200 mg/kg) produced a significant decrease in serum TC and LDL-C levels in HFD-fed mice. Serum ALT and LDH levels (hepatic damage markers) increased significantly in the HFD group compared with those in the control group. Treatment with LJE reduced these increases in hepatic enzyme levels significantly to values comparable to those in the control group. Serum MDA levels, an oxidative marker, increased significantly, by 80%, in the HFD group compared to the control group. However, LJE reduced the serum MDA levels in HFD-fed mice significantly (by 60% and 80%, respectively).

### 3.7. Effects of LJE on Serum Glucose and Insulin in HFD-Fed Mice 

**Figure 6:**
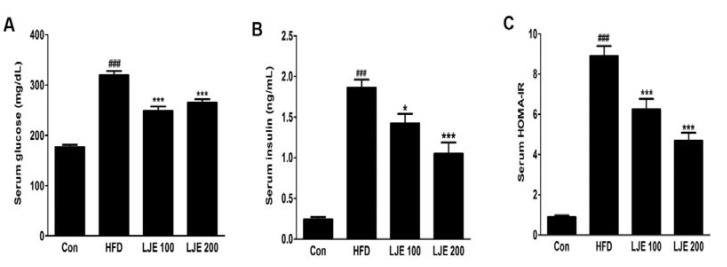
Effects of LJE on serum glucose, insulin, and homeostasis model assessment-insulin resistance (HOMA-IR) in HFD-fed mice. (**A**) Serum glucose levels; (**B**) Serum insulin levels; (**C**) Serum HOMA-IR. Con, normal control diet; HFD, high-fat diet; LJE 100, HFD plus LJE (100 mg/kg, peroral); LJE 200, HFD plus LJE (200 mg/kg, peroral). HOMA-IR, homeostasis model assessment values for insulin resistance. Data represent means ± SEM (*n* = 8). ^###^ *p* < 0.001 vs. Con; * *p* < 0.05, *** *p* < 0.001 vs. HFD.

The HFD-group showed marked increases in fasting serum glucose and insulin levels compared with those in the control group (Figure 6A,B). Furthermore, the HOMA-IR index increased by about 10-fold in the HFD group compared with the control group (Figure 6C). However, LJE treatment (100 or 200 mg/kg) reduced the fasting serum glucose and insulin levels significantly (Figure 6A,B). Insulin sensitivity, as assessed by HOMA-IR, was also reduced significantly in the LJE-treated groups compared with the HFD group (by 30% or 47%, respectively; Figure 6C). These results indicated that LJE regulates glucose levels by improving insulin resistance in HFD-fed mice.

### 3.8. Effects of LJE on the Liver Steatosis in HFD-Fed Mice

**Figure 7:**
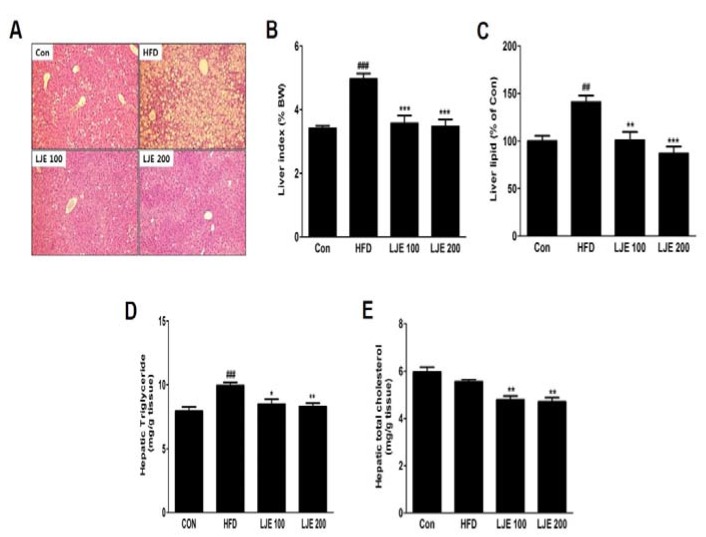
Effects of LJE on liver steatosis in HFD-fed mice. (**A**) Liver sections stained with hematoxylin and eosin (100×); (**B**) Liver index; (**C**) Liver lipid content; (**D**) Hepatic triglyceride levels; (**E**) Hepatic total cholesterol levels. Con, normal control diet; HFD, high-fat diet; LJE 100, HFD plus LJE (100 mg/kg, peroral); LJE 200, HFD plus LJE (200 mg/kg, peroral). Data represent means ± SEM (*n* = 8). ^##^ *p* < 0.01, ^###^ *p* < 0.001 vs. Con; * *p* < 0.05, ** *p* < 0.01, *** *p* < 0.001 vs. HFD.

A major cause of NAFLD is liver fat accumulation during ingestion of HFD [18]. Histological analysis showed significant increases in lipid droplets in hepatocytes in the HFD group, whereas these lipid droplets were decreased significantly, in both size and numbers, in LJE-treated groups (Figure 7A). Consistent with the histological data, the relative liver weights (liver index), liver lipid contents, and liver TG levels in the HFD group were significantly increased (by 45%, 41%, and 25%, respectively) compared with those in the control group (Figure 7B–D). LJE administration (100 or 200 mg/kg) improved hepatic steatosis and hyperlipidemia significantly by reducing liver lipid, TG, and TC levels in the HFD-fed mice (Figure 7A–E). 

### 3.9. Effects of LJE on Hepatic Lipid Metabolism in HFD-Fed Mice

**Figure 8:**
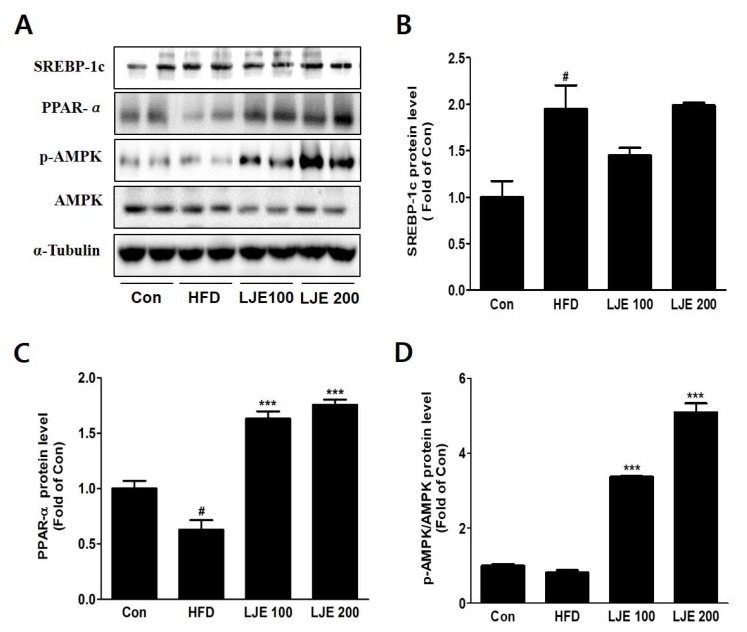
Effects of LJE on hepatic lipid metabolism in HFD-fed mice. (**A-D**) SREBP-1, PPAR-α, and phosphorylated AMPK protein levels by immunoblot analysis. Con, normal control diet; HFD, high-fat diet; LJE 100, HFD plus LJE (100 mg/kg, peroral); LJE 200, HFD plus LJE (200 mg/kg, peroral). Data represent means ± SEM (*n* = 8). ^#^ *p* < 0.05 vs. Con; *** *p* < 0.001 vs. HFD.

To investigate the molecular mechanisms, we assessed protein expression during hepatic lipogenesis by Western blotting. SREBP-1c protein levels increased significantly in the HFD group compared with the control group. There were no significant differences in the LJE-treated groups (Figure 8A,B). Furthermore, LJE treatment of HFD-fed mice caused dose-dependent increases in fatty acid oxidation-related gene expression as well as PPAR-α and phospho-AMPK levels (Figure 8C,D).

## 4. Discussion

Recently, the prevalence of NAFLD has increased rapidly, together with the dramatic increases in obesity and metabolic syndrome worldwide [19]. Obesity-associated NAFLD shows various features, including hepatic steatosis, insulin resistance, hyperlipidemia, and fat droplet deposits in the liver. 

In this study, we investigated the underlying mechanisms involved in the therapeutic efficacy of LJE to treat NAFLD, using an in vitro model involving treatment with FFA-loaded HepG2 cells and an in vivo model using HFD-fed mice, partly through regulating the gene expression involved in lipid metabolism. Our results demonstrated that LJE treatment significantly inhibited lipid accumulation and intracellular TG levels in a concentration-dependent manner in FFA-treated HepG2 cells. Consistent with these findings, a previous study stated that leonurusoides from the aerial parts of *L. japonicus* inhibited TG accumulation in HepG2 cells treated with 0.2 mM oleic acid [20]. 

Consistent with the in vitro experiments, HFD-fed mice showed significant increases in body weight and liver indexes, which induced obesity. However, LJE treatment for 14 weeks significantly decreased the body weight, serum ALT, LDH, and MDA levels induced by HFD to values similar to those of the control, suggesting that LJE exerts anti-obesity effects. 

These results were consistent with previous reports that chronic intake of an HFD induced liver damage and increased serum ALT and MDA, which are used as surrogate markers of NAFLD [21,22]. 

The HFD group also showed characteristics of fatty liver disease, including increased hepatic lipid content, hepatic TG levels, and fat droplet vacuoles in the liver. Consistent with the results using the in vitro model in HepG2 cells, LJE supplementation significantly eliminated hepatic lipid accumulation in the HFD-fed mice. These results suggested that LJE could represent a new therapeutic drug to treat obesity-induced hepatic steatosis.

According to the theory of NAFLD pathogenesis, fat accumulation in hepatocytes caused by insulin resistance (IR) and lipid metabolism disorders results in hepatocellular fat degeneration [23]. In the present study, the HFD group demonstrated a significant increase in serum glucose, insulin levels, and HOMA-IR, which indicated a higher degree of IR. However, LJE treatment ameliorated hyperglycemia and elevated HOMA-IR associated with HFD. Aqueous or methanolic extract of *L. japonicus* could stimulate the proliferation of insulinoma INS-1E cells, suggesting that *L. japonicus* could enhance insulin secretion and could be applied as a herbal medicine to treat diabetes and related disorders [24]. 

IR promotes fatty acid synthesis and gluconeogenesis, which further contribute to the development of dyslipidemia, hyperglycemia, and type 2 diabetes [25]. Excessive hepatic lipid accumulation is a characteristic of NAFLD, which is caused by an imbalance between fatty acid synthesis and fatty acid oxidation [26,27]. In patients with NAFLD, high concentrations of circulating FFAs might stimulate hepatic lipid accumulation by interfering with lipid metabolism. 

To determine the mechanisms by which LJE regulates lipid metabolism, we investigated gene expression related to de novo lipogenesis and fatty acid oxidation. Hepatic steatosis might be mediated partly by regulating SREBP-1c, a key transcription factor of lipogenic genes in fatty acid synthesis and TGs, including *ACC*, *SCD*, and *FAS* [28,29]. Overexpression of SREBP-1c induced a fatty liver because of increased lipogenesis in HFD-induced obesity and IR diabetes models [30]. CD36 mediates the uptake of FFAs across the cell membrane, and its high expression is usually associated with IR and type 2 diabetes, including HFD-induced obesity [31]. We observed that LJE markedly reduced the gene expression involved in lipogenesis in FFA-induced HepG2 cells. 

During lipid metabolism, phosphorylation activation of AMPK suppresses hepatic lipogenesis by inactivating ACC, which is its primary downstream target enzyme [32,33]. Indeed, activation of the AMPK pathway decreased phospho-ACC/ACC, as well as several other lipogenesis-related proteins [34]. AMPK plays a pivotal role in mediating the expression of factors involved in fatty acid oxidation, such as PPARα and CPT-1 [35]. PPARα prevents hepatic fat deposition by stimulating fatty acid catabolism. CPT-1 is the rate-limiting enzyme in fatty acid oxidation and regulates the inflow acyl-CoA and β-oxidation in the mitochondrial outer membrane [36]. The regulatory function of AMPK could represent a therapeutic target to treat NAFLD [37]. Moreover, we demonstrated that LJE increased the phosphorylation of AMPK and the mRNA expression of *PPARα or CPT-1* in FFA-induced HepG2 cells. Consistent with our in vitro findings, treatment of HFD-fed mice with LJE suppressed lipogenesis in the liver by increasing the levels of phospho-AMPK and PPARα. These results suggested that LJE exerts its lipid-lowering effects via the modulation of transcript levels of *SREBP-1c* and *PPARα*, and modulators for lipogenesis and lipolysis, respectively. Therefore, LJE treatment could have a therapeutic effect on NAFLD models; i.e., FFA-induced HepG2 cells and HFD-induced obese mice.

## 5. Conclusions

This study showed that LJE inhibited lipid accumulation in FFA-induced HepG2 cells significantly. In HFD-induced obese mice, LJE decreased body weight gain; serum ALT, MDA, glucose and insulin levels; hepatic lipids; and hepatic TG. Furthermore, LJE downregulated the expression levels of lipogenesis genes including *SREBP-1*, *FAS*, *SCD-1*, and *CD36*, and upregulated the expression levels of fatty acid oxidation genes including *AMPK*, *PPARα*, and *CPT-1*. Taken together, LJE could prevent hepatic steatosis by suppressing lipogenesis and might be considered a useful treatment for NAFLD. 

## Figures and Tables

**Table 1:** Primer sequences used in quantitative real-time PCR.

Gene	Primers	Sequence (5′ to 3′)
*SREBP-1c*	Forward	TGCATTTTCTGACACGCTTC
	Reverse	CCAAGCTGTACAGGCTCTCC
*FAS*	Forward	CCCCTGATGAAGAAGGATCA
	Reverse	ACTCCACAGGTGGGAACAAG
*SCD-1*	Forward	TTGGAGAAGCGGTGGATAAC
	Reverse	AAAAATCCCACCCAATCACA
*CD36*	Forward	TGGAACAGAGGCTGACAACT
	Reverse	TTGATTTTTGATAGATATGGG
*PPAR-α*	Forward	ACGATTCGACTCAAGCTGGT
	Reverse	GTTGTGTGACATCCCGACAG
*CPT-1*	Forward	CCT CCG TAG CTG ACT CGG TA
	Reverse	GGA GTG ACC GTG AAC TGA AA
*β-actin*	Forward	CTCTTCCAGCCTTCCTTCCT
	Reverse	AGCACTGTGTTGGCGTACAG

SREBP-1c, sterol regulatory element binding protein1-c; FAS, fatty acid synthase; SCD-1, stearoyl-CoA desaturase-1; CD36, cluster of differentiation 36; PPAR-α, peroxisome proliferator-activated receptor-alpha; CPT-1, carnitine palmitoyltransferase-1.

**Table 2:** Effects of LJE on serum biochemical parameters in HFD-fed mice.

	Con	HFD	LJE 100	LJE 200
TC (mg/dL)	165.63 ± 10.50	232.50 ± 18.10 ^###^	188.33 ± 27.87 **	179.29 ± 23.88 ***
LDL-C (mg/dL)	20.44 ± 1.24	25.93 ± 3.60 ^##^	22.58 ± 2.35 *	19.08 ± 2.85 ***
TG (mg/dL)	114.17 ± 10.21	113.33 ± 15.71	116.25 ± 9.16	107.50 ± 9.64
AST (IU/L)	113.00 ± 34.21	155.00 ± 39.79	120.00 ± 32.79	95.63 ± 26.92 *
ALT (IU/L)	81.43 ± 26.73	215.00 ± 40.74 ^###^	106.43 ± 47.58 **	92.86 ± 40.50 ***
LDH (IU/L)	570.00 ± 182.30	872.86 ± 113.94 ^##^	628.75 ± 171.85 *	524.29 ± 120.33 **
MDA (μM)	1.05 ± 0.26	1.91 ± 0.25 ^###^	1.30 ± 0.36 *	1.10 ± 0.53 **

Data represent means ± SEM (*n* = 8); Con, normal control diet; HFD, high-fat diet; LJE 100, HFD plus LJE (100 mg/kg, peroral); LJE 200, HFD plus LJE (200 mg/kg, peroral). ^##^ *p* < 0.01, ^###^ *p* < 0.001 vs. Con. * *p* < 0.05, ** *p* < 0.01, *** *p* < 0.001 vs. HFD.
